# Overview of Patagonian Red Octopus (*Enteroctopus megalocyathus*) Fisheries in Chilean Regions and Their Food Safety Aspects

**DOI:** 10.3390/ani15101464

**Published:** 2025-05-19

**Authors:** Alessandro Truant, Federica Giacometti, Jorge Hernández, Viviana Espinoza, Ana Farías, Iker Uriarte, Cecilia Godoy, Riccardo Miotti Scapin, Leonardo Alberghini, Paolo Catellani, Valerio Giaccone

**Affiliations:** 1 Department of Animal Medicine, Production and Health, University of Padova, Legnaro, 35020 Padova, Italy; alessandro.truant@phd.unipd.it (A.T.); riccardo.miottiscapin@unipd.it (R.M.S.); leonardo.alberghini@unipd.it (L.A.); paolo.catellani@unipd.it (P.C.); valerio.giaccone@unipd.it (V.G.); 2 Hatchery of Marine Invertebrates, Institute of Aquaculture and Environment, Universidad Austral de Chile, Valdivia 5110566, Chile; jorge.hernandez@uach.cl (J.H.); viviana.espinoza@uach.cl (V.E.); afarias@uach.cl (A.F.); iuriarte@uach.cl (I.U.); 3 Fundación Bordemar, Puerto Varas 5550000, Chile; mcgodoya@gmail.com

**Keywords:** *Enteroctopus megalocyathus*, artisanal fisheries, food safety, territorial use rights in fisheries (TURFs), sustainability

## Abstract

Artisanal fisheries in southern Chile rely heavily on the Patagonian red octopus (*Enteroctopus megalocyathus*) as a valuable resource, contributing significantly to local economies. This octopus species accounts for 25–40% of Chilean octopus landings. It is a merobenthic species, characterized by a semelparous life cycle and a long brooding period, and it is distributed along the Pacific and Atlantic coasts of the southern tip of South America, inhabiting holes and crevices in rocky substrates. However, this fishery faces critical challenges to both its ecological sustainability and the food safety of octopus products. The primary fishing method, using hooks, poses a risk to reproductive capacity as it can capture brooding females. Food safety concerns arise from microbial contamination during pre- and post-harvest handling, bioaccumulation of toxins from algal blooms, and the presence of heavy metals in the marine environment. While evisceration effectively reduces the risk of consuming toxins and heavy metals, inadequate hygiene practices and insufficient ice usage throughout the production chain represent significant food safety risks. Chilean fishing Law No. 18892/1989 defines artisanal fishing and establishes territorial use rights in fisheries (TURFs) to promote sustainable extraction of benthic resources. Integrating training programs on post-harvest handling, hygiene practices, and food safety measures into the TURFs framework, along with targeted investments in infrastructure and technical assistance, is crucial to ensure the long-term sustainability of the *E. megalocyathus* fishery, protect consumer health, and maintain the economic viability and environmental sustainability of this vital resource for local communities.

## Simple Summary

This study examines the Patagonian red octopus (*Enteroctopus megalocyathus*) fisheries in southern Chile, highlighting the significant socioeconomic role of the species in artisanal fishing communities amidst significant challenges to ecological sustainability and food safety. Key issues include overfishing risks from current fishing practices, such as hook fishing, which can capture brooding females, and inadequate enforcement of management measures. Food safety concerns arise from microbial contamination during handling and bioaccumulation of algal toxins and heavy metals, although evisceration reduces some risks. Particularly problematic are insufficient hygiene and lack of cold chain maintenance in remote artisanal areas. This study discusses existing management tools like territorial use rights in fisheries (TURFs) and proposes integrating training programs on post-harvest handling and food safety, investing in infrastructure and technical assistance within TURFs, and exploring small-scale aquaculture. These interventions are considered crucial for ensuring fishery’s long-term viability, protecting consumer health, and supporting local livelihoods under a One Health approach.

## 1. Introduction

A growing global concern is related to the overexploitation of marine resources and consequent impact on marine ecology as well as the availability of high nutritional protein sources for human consumption [1]. On the one hand, the global overexploitation and depletion of many finfish species over the last few decades has led to an increase in the commercial importance of other marine resources, such as cephalopods (squids, cuttlefishes, and octopuses) [2]. On the other hand, the negative trend of overexploitation refers to different fish species and fishing areas, including marine carnivorous mollusks, namely cephalopods [3]. Among cephalopod landings, the squid fishery represents nearly 80% of the worldwide catches, whereas octopuses and cuttlefishes represent only about 10% each [4]. Octopus fisheries are likely to continue to grow in importance and magnitude as many finfish stocks are either fully or overexploited [4]. Fishery stocks that are scientifically assessed are in better condition, either already under sustainable exploitation or rebuilding, than stocks that are unassessed [5]. The vast majority of stocks are unassessed [6], especially stocks exploited by small-scale fisheries.

**Scheme 1:**
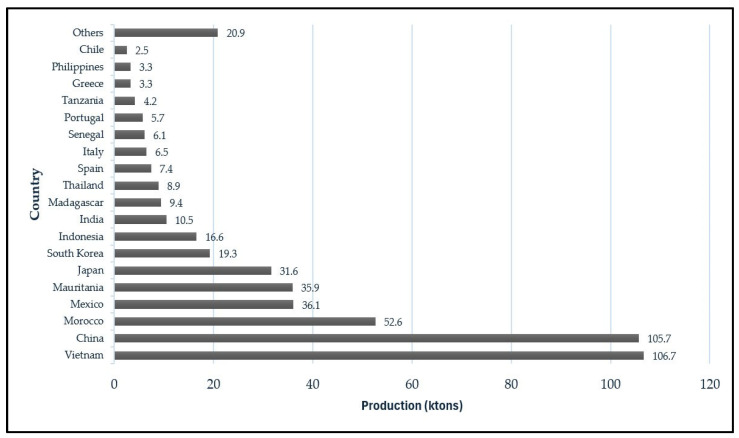
Average global octopus production (2019–2021, adapted from Di Todaro [16]).

In the specific case of Latin America, octopus fisheries represent only about 8% of cephalopod world catches (see Scheme 1 for more details). This is mainly due to the recent development and the continued dominance of artisanal, small-scale, and low-productivity fishing activities (except for the well-developed octopus fishing sector in the Campeche Bank of the Yucatan Peninsula) [7]. Contributing factors include limited investment in fisheries research and development, limited technical development of artisanal fleets, a lack of specific public policies for the sustainable management of the resource, and weak integration of regional markets into international cephalopod value chains. Furthermore, many Latin American countries prioritize other, more established fisheries (such as anchovy, shrimp, and hake), relegating octopus to a secondary role. Additional challenges include logistics, traceability, and compliance with international regulations that limit its commercial expansion. Nevertheless, there are emerging initiatives led by local communities, research institutions, and certain government bodies aimed at enhancing the ecological and socioeconomic value of octopus resources, potentially paving the way for greater participation in global markets in the future [8,9,10]. Octopus catches in the Eastern Pacific are mainly taken by Chile, Peru, and Mexico, but Octopus is also common in the Southeast Pacific coast with a wide oceanic and coastal distribution [7]. In the southern tip of South America (Argentina and Chile), the octopus fishery is largely based on *Enteroctopus megalocyathus*, the Patagonian red octopus, a merobenthic species that inhabits the Pacific and Atlantic coasts, from 42° S to the Strait of Magallanes, including the Malvinas/Falkland Islands [11]. Specifically, in southern Chile, *E. megalocyathus* is an important resource representing a high value product with significative importance for local economies in several areas where small-scale fisheries, namely artisanal fisheries, are still in practice today. This phenomenon can be observed in southern Chilean regions, such as the Los Lagos region, where local communities of fishers (36,556 fishers recorded in 2023) base their familiar economy on artisanal fishing activities and for which local octopus species play an important role [12]. *Robsonella fontaniana*, commonly referred to as “pulpito” or “baby octopus”, is another species frequently caught in the intertidal waters of the Los Lagos region due to its overlapping geographical distribution with *E. megalocyathus*. This species exhibits a broad distribution range, extending from approximately 41° S along the Pacific and Atlantic coasts to 56° S near Cape Horn, Chile [13]. Economically, it remains a marginal species with no support for targeted commercial exploitation, and to date, no official landing records are available [4].

Studies on *E. megalocyathus* from the Atlantic and Pacific oceans have increased notably in the last 20 years, mostly driven by its relevance as a fishery resource and growing interest as a culture species [14]. However, important biological and ecological aspects of this species remain to be investigated, as well as food safety aspects for consumers. Comprehensive investigations and a deeper understanding of the structure and dynamics of natural populations are needed, particularly during the early developmental stages, where knowledge of paralarval ecology remains limited. Clarifying the spatial and temporal occurrence of early life stages, and elucidating their interactions with biotic and abiotic factors, is critical for advancing our understanding of paralarval dispersal, recruitment processes, and the associated interannual variability in cephalopod population biomass [15]. Promoting research focused on habitat selection and utilization throughout the ontogeny of the red octopus, as well as assessing physiological and ecological responses to environmental drivers, is essential, particularly under ongoing climate change scenarios. Additionally, detailed insights into the trophic ecology of the red octopus and its functional role within marine food webs are still lacking, representing a key knowledge gap that should be addressed. This paper describes the *E. megalocyathus* fisheries in Chilean waters, providing an update review on fisheries, including artisanal fisheries, in the Los Lagos region, with the aim of detailing general food safety aspects in octopus fisheries and products, based on the available literature and public domain data.

## 2. Biology and Fishery Exploitation of the Patagonian Red Octopus (*Enteroctopus megalocyathus)*

The Patagonian red octopus (*E. megalocyathus*) represents a species of considerable ecological and socioeconomic importance across the southern coastal ecosystems of Chile and Argentina. As a key component of benthic communities and a valuable target for artisanal fisheries, *E. megalocyathus* plays a pivotal role in sustaining both biodiversity and local livelihoods. However, comprehensive understanding of its biology, life history traits, and ecological interactions remains limited, particularly with respect to early developmental stages, habitat preferences, and trophic dynamics. Increasing fishing pressure over the past decades has raised significant concerns regarding stock sustainability, leading to the implementation of management measures such as seasonal closures and the establishment of territorial use rights in fisheries (TURFs). A thorough characterization of the species’ biological and ecological attributes, alongside an analysis of artisanal fishing practices and regulatory frameworks, is thus essential to inform effective conservation strategies and promote the long-term sustainability of *E. megalocyathus* fisheries. The following sections provide an integrated overview of these aspects, highlighting both current knowledge and persisting gaps that require further research.

### 2.1. Distribution, Life Cycle, and Capture

#### 2.1.1. Geographical Distribution

**Figure 1:**
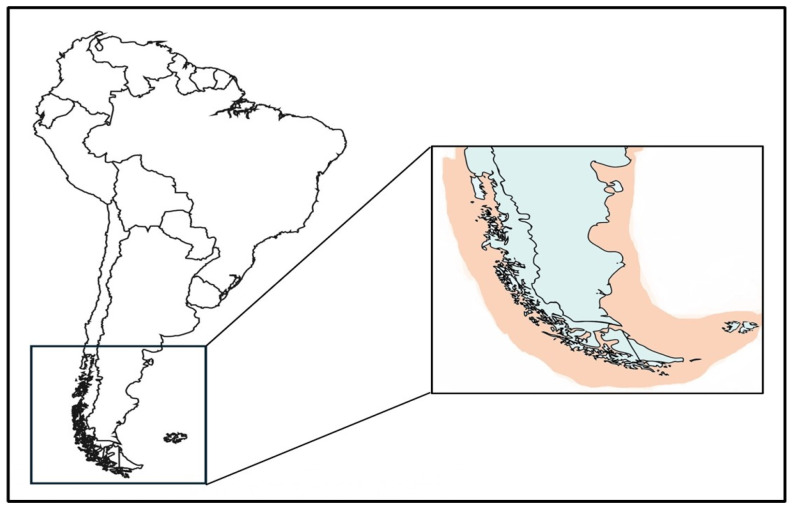
*Enteroctopus megalocyathus* geographical distribution (authors’ own elaboration).

The Patagonian red octopus, *E. megalocyathus* (Couthoy in Gould, 1852), is one of the giant octopuses (genus *Enteroctopus*, Family Enteroctopodidae) and can reach up to 7 kg wet weight, over 30 cm mantle length, a total length of 1.3 m [17], and a lifespan between 1.9 and 2.5 years [18]. It is the main octopus species present in the southern Chilean and Argentinean coasts. In more detail, *E. megalocyathus* is distributed from the Los Lagos region (41° S, 72°00′ W) throughout the Patagonian Shelf around the southern coast of South America from the Magellan biogeographic province, from Chiloe Island in the Southeast Pacific, via Cape Horn, to the San Matías Gulf in the Southwest Atlantic. It includes the Beagle Channel, Malvinas/Falkland Islands, and Namuncurá (Burdwood) Bank [17,19,20] (Figure 1). The Pacific coast, from north of Chiloe Island to Cape Horn, is described as a large insular system (the Chilean Archipelago) characterized by numerous gulfs, fjords, channels, small river basins, and many conspicuous glaciers, which are the product of tectonic processes and ice ages. This makes it a complex landscape with difficult access both by land and sea [21]. The Patagonian red octopus occupies holes and crevices in rocky substrates from the lower intertidal zone down to a depth of 170 m off Argentina and down to a depth of 220 m off Chile [17]. Within its distribution, there are two genetically distinct *E. megalocyathus* populations, one on the Pacific coast and the other on the Atlantic coast, with low genetic diversity in contrast to the high variability found in other octopus species (*Octopus variabilis, Octopus minor, Octopus vulgaris*, and *Amphioctopus ovulum*) [17].

#### 2.1.2. Life Cycle and Trophic Ecology

**Figure 2:**
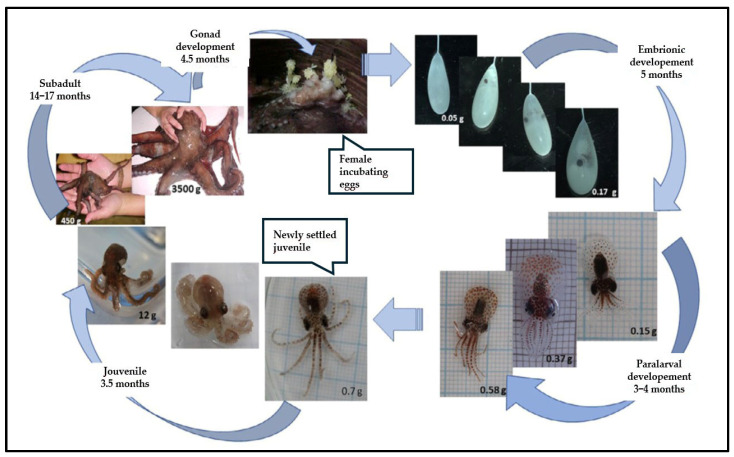
*Enteroctopus megalocyathus* life cycle (figure adapted from Ortiz et al. [17] with permission from the authors).

Like other cephalopods, it is a short-living semelparous organism with a seasonal breeding activity. Individuals exhibit separate sexes with marked sexual dimorphism, with males displaying a modified arm called the hectocotylus, which is used for spermatophore transfer to females (a single case of hermaphroditism has been reported in this species) [22,23]. The sexual maturity of individuals starts in winter, reaching its maximum in spring, with the peak of reproductive activity in summer, between December and January [20]. Sexual maturity is mainly influenced by individual size. After mating and breeding activities, which usually takes places in deeper locations than fishing areas [24], females lay egg strings, sticking them in the roofs of rock holes from 6 to 14 m depth [25]. The eggs are highly vulnerable to predation, and the female provides continuous protection, cleaning, and ventilation throughout the entire developmental period, at the end of which they will die [26]. After 5 months, the paralarvae of Patagonian red octopuses will hatch [11]. This phase is characterized by planktonic paralarvae that feed on different planktonic microorganisms, which lasts about 90 to 114 days, before moving to the benthic phase as juveniles (Figure 2).

It is not known if, in this life stage, paralarvae remain in coastal areas or migrate into the open ocean, transported by sea currents (as suggested for other species by Roura et al. [27]), until, after 3–4 months, they reach the juvenile stage, adopting the classical benthic behavior [28]. Concerning bathymetric distribution, adult *E. megalocyathus* can be found at different depths depending on environmental temperatures. With water temperatures between 12 and 13 °C, individuals are usually found between 15 and 30 m depth along the Pacific coast, whereas they occur at depths of less than 20 m along the Atlantic coast [18]. When higher temperatures (>13 °C) are present, adult benthic individuals have been found at depths of up to 170 m [24], with the record at 220 m depth [29]. A study conducted by the Chilean Instituto de Fomento Pesquero (IFOP) reported environmental parameters characterizing the habitat of *E. megalocyathus* along the coasts of Chiloe Island, highlighting water temperatures between 9.8 and 13.5 °C, salinity ranging from 32.1 to 33.4 PSU, and oxygen concentrations between 8.4 and 9.2 mL-L^−1^ (with saturation levels reaching up to 100%) [23].

*E. megalocyathus* is an opportunistic predator with a broad prey spectrum. Although it presents wide dietary variability, for both the Pacific and Atlantic populations, the diet is predominantly based on decapod crustaceans, mainly from the Order Brachyura (*Homalaspis plana*, *Cancer setosus*, and *Cancer coronatus*) and the Order Anomura (*Petrolisthes* sp., *Munida subrugosa*, and *Peltarion spinosulum*) [18,30,31]. Several studies have suggested that octopus predation may play a significant role in shaping benthic community structure [32,33]. With high voracity, broad dietary spectrum, and substantial population density, *E. megalocyathus* likely acts as a key predator in both intertidal and subtidal ecosystems, potentially exerting a notable influence on community composition and trophic interactions [17]. Cannibalistic behavior has also been reported, particularly during early developmental stages when paralarvae density is high and/or prey availability is low [34]. On the other hand, the main predators of the red octopus include Peale’s dolphin (*Lagenorhynchus australis*) [35], spiny dogfish (*Squalus acanthias*) [36,37], South American sea lion (*Otaria flavescens*) [38], western rockhopper penguin (*Eudyptes chrysocome*) [39,40], gentoo penguin (*Pygoscelis papua*) [41], and humans (fishers). A more comprehensive overview of the principal red octopus preys and predators is presented in Table 1. All of these environmental factors affect the octopus life cycle, thus affecting population dynamics and densities in certain areas throughout the different seasons.

#### 2.1.3. Landings and Fishery Relevance

In relation to abundance, the total reported global production of octopuses over the past three decades indicates a relatively steady increase in catch, almost doubling from 179,042 tonnes in 1980 to 355,239 tonnes in 2014 [4]. In Chile, *E. megalocyathus* is a native and merobenthic species; considering its high nutritional, organoleptic value and massive presence in coastal areas, an intensive local exploitation for food consumption of this marine resource is reported. *E. megalocyathus* was determined to be the second most important octopus species after *Octopus mimus,* although its abundance data were not clear and difficult to compare. Reconstructed data reported an average of 700 tonnes for landings by year, with a catch peak at 1700 tonnes in 2008, whereas octopus catches by other countries in the region are trivial [7]. For 2008, an average landing of 500 tonnes/year of *E. megalocyathus* in Chilean water is always reported [20]. Between 2009 and 2011, a complete fishing ban was imposed on this species due to overexploitation. Subsequently, a seasonal fishing ban was implemented, restricting fishing activities from 15 October to 15 March each year, to support the species’ reproductive cycle, along with a decrease in the minimum fishing weight of 1 kg (Supreme Decree No. 137/1985) [59]. In 2016, a total of 540 tonnes (equivalent to 0.7% of the global octopus production) of the Patagonian red octopus were landed in Chile [60]. *E. megalocyathus* accounts for 25–40% of Chilean octopus landings [17]. In 2023, an annual landing of red octopus of 571 tonnes was reported in the last available SERNAPESCA report record as “Pulpo del Sur” [13]. Almost 100% of *E. megalocyathus* catch is landed in the Los Lagos region. It reaches its highest population densities and optimal commercial sizes in the cool–temperate waters of the Los Lagos region (Region X) and Aysén (Region XI), where it finds ideal habitats such as rocky bottoms and coastal caves. The water temperature in this region remains within a favorable range for the growth and reproduction of *E. megalocyathus*. These habitats are easily accessible for small-scale fishing (with apnea or traps), without the need for expensive vessels or extensive technology. In the Los Lagos region, there is also a large concentration of artisanal fishers organized in management areas, which promotes a more planned and legal use of the resource.

**Scheme 2:**
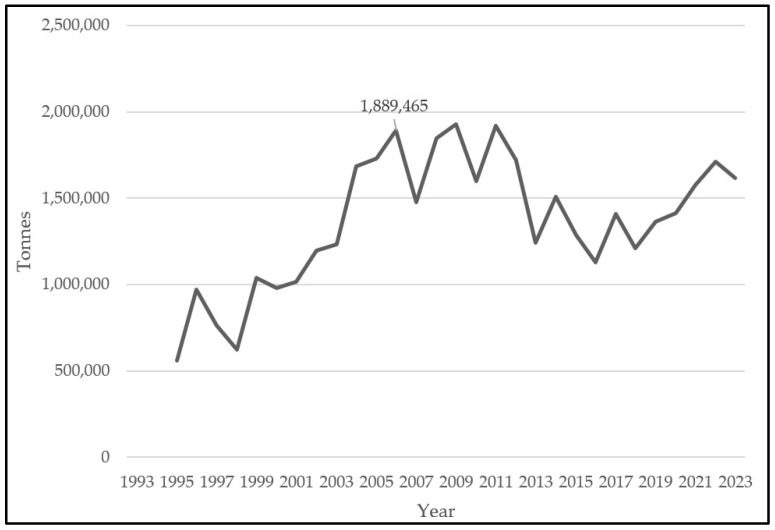
Total artisanal landings in Chile (1995–2003, authors’ own elaboration based on public domain data from SERNAPESCA).

**Scheme 3:**
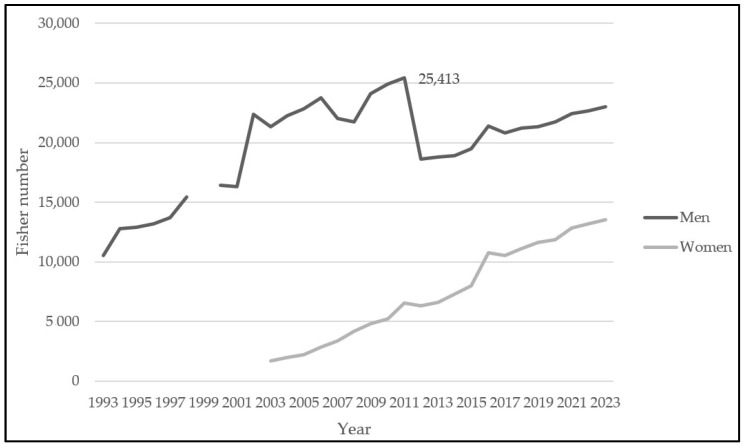
Number of fishers registered in the Artisanal Fishery Register (Registro Pesquero Artesanal, RPA; authors’ own elaboration based on public domain data from SERNAPESCA).

In Chile, a total of 375 small-scale fisheries are targeting invertebrates and macro-algae. Total artisanal fishing landings and number of artisanal fishers showed an increasing trend over recent years (Scheme 2 and Scheme 3). The catch of *E. megalocyathus* is carried out by divers that travel to numerous fishing grounds in small, motorized boats. The octopus season extends from March to October. The data for stock assessment and total annual landings were collected in Chile by the “Servicio Nacional de Pesca y Acuicultura” (SERNAPESCA), which carries out a census of all fishing trips to evaluate the magnitude of the catch for all large-scale fisheries and for the most important small-scale fisheries. In the case of the red octopus of the Inner Sea of Chiloe, fishers have not yet learned about the sustainable exploitation rates because the fishery is too young, and thus they entered a regime of over-fishing by the time of the end of the study reported in [5]. Indeed, as clearly described by Markaida and Gilly [7], in southern Chile, an overfishing of *E. megalocyathus* has occurred and, hitherto, small scale octopus fisheries are experiencing different challenges. In fact, even small-scale fisheries like those for *E. megalocyathus* have been interrupted or banned for some years due to overfishing concerns [11,19]. Open access to the fishery by non-fisher divers with hooks and use of gear such as pots that retain spawning females are current concerns in the octopus fisheries; moreover, improper identification of commercial species is still a common problem [61]. In addition, management measurements such as minimum size or gear restrictions are difficult to enforce in these fisheries. Therefore, there are still areas of opportunity, such as developing methods of octopus fisheries management [7].

## 3. Management Strategies for the Patagonian Red Octopus (*Enteroctopus megalocyathus*)

### 3.1. Artisanal Fishing Laws in Chile

**Scheme 4:**
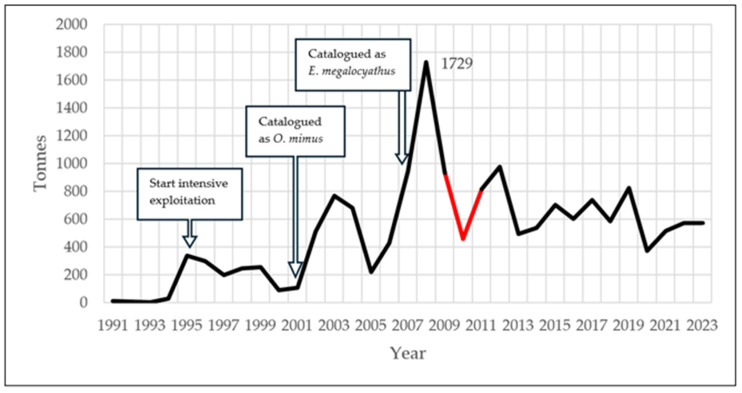
*Enteroctopus megalocyathus* artisanal landings in the Los Lagos region (authors’ own elaboration based on public domain data from SERNAPESCA). The red line indicates the fishing ban period (2009–2011). The reported catches came from an exceptional derogation of the fishing ban.

Article No. 28 of Chilean fishing Law No. 18892/1989 (“Ley General de Pesca y Acuicultura”) [62] defines artisanal fishing as the extractive fishing activity carried out by natural persons in a personal, direct, and habitual way, and in the case of territorial use rights in fisheries (TURFs), by legal entities composed exclusively of artisanal fishers registered as such. Employees on fishing boats used for artisanal fishing are considered artisanal fishers. This definition also includes extractive fishing divers, shore collectors, and algae collectors (Article No. 29 of Chilean fishing Law No. 18892) [62]. This means that artisanal fishing is characterized by a wide spectrum of different activities, which can range from the recollection of costal resources to scuba diving, with different techniques, different boats, and different roles of the fishers, and it accounts for a substantial share (39% in 2023) of Chile’s total fisheries output (considering both fishing landings and aquaculture production) [63]. For local fishers, artisanal fishing is a traditional and hereditary activity with a strong socio-cultural component. Artisanal fisheries provide fundamental socioeconomic support to rural coastal communities in Chile. Small-scale fishing activities represent a primary source of employment, income, and food security, generating significant revenues for fishers [64]. The legal requirement to become an artisanal fisher is inscription in the National Fishing Register, which shows an annual increasing trend of new inscriptions (Registro Pesquero Artesanal, RPA). A total of, respectively, 58,873, 81,157 (+37.8%), and 89,697 (+10.5%) fishers were registered in 2000, 2010, and 2018 [65,66,67]. At the end of 2023, a total of 103,017 artisanal fishers (26% of which are female) were reported [68] (Graph Y). All of these fishers are organized into different associations, like the local fisher organizations, namely the National Confederation of Artisanal Fishermen of Chile (Confederación Nacional de Pescadores Artesanales de Chile), the National Confederation of Artisanal Fishermen Federations (Confederación Nacional de Federaciones de Pescadores Artesanales), and the National Council for the Protection of Fish Stocks (Consejo Nacional por la Defensa del Patrimonio Pesquero). At the end of 2023, in the Artisanal Organization Register (Registro de Organizaciones Artesanales), a total of 1850 different fisher associations (considering federations, confederations, syndicate, trade associations, and indigenous communities) were registered. The creation of fisher associations gives social, political, and economic strength to artisanal fishers, obtaining better results in the relationship with institutions. Improper identification of commercial species is still a common problem. In southern Chile, this has led to the overfishing of *E. megalocyathus*. A ban had to be implemented, and Chilean statistics now discriminate octopus landings by species (since 2010). This emphasizes the need to accurately identify any species being landed [7]. In the Los Lagos region, almost 571 tons of Patagonian red octopus were landed in 2023 [13] (Scheme 4).

According to official reports from SERNAPESCA, *E. megalocyathus* and *O. mimus* were historically recorded as a single fishery resource until 2007. Beginning in 2008, official statistics began to differentiate between the two species, listed, respectively, as “Pulpo del Sur” (southern octopus) and “Pulpo del Norte” (northern octopus). *O. mimus* is distributed along the northern coast of Chile (2.2–27.7° S), with no geographic overlap with *E. megalocyathus* [69]. Based on this, landing data from the Los Lagos region in SERNAPESCA reports are interpreted as pertaining exclusively to *E. megalocyathus*. Given the historical absence of a systematic identification protocol, a comparative table is provided to highlight the diagnostic features useful for species differentiation (Table 2).

### 3.2. Territorial Use Rights in Fisheries (TURFs), the Management Areas for Benthic Resource Exploitation

Article No. 47 of Chilean fishing Law No. 18892/1989 [62] identifies the first 5 miles from the coast as an exclusive area for artisanal fishing activities (“Área de reserva para la pesca artesanal”, ARPA), while industrial fishing activities are allowed in the open sea. Artisanal fishing activities are carried out mainly in coves, called “Caletas”, which are the productive, economic, social, and cultural centers in which this fishing sector originates. At the end of 2023, a total of 558 active “Caletas” were reported all along the Chilean coast [70]. In this area, Article No. 55 of Law No. 18892/1989 gives the possibility to legal fisher associations to apply for the creation of territorial use rights in fisheries (TURFs) (“Areas de manejo y explotación de recursos bentónicos, AMERB”, textually translated as “Management areas for the exploitation of marine benthic resources”). This is an administrative and management measure to give exploitation rights of benthic resources to local artisanal fisher associations. In these TURFs, artisanal fishers can extract resources from the sea bottom, which are identified as main species in Chilean law. All the extracted products are part of fishing quotas related to management plans of that specific coastal area, which must be approved by Undersecretariat of Fisheries and Aquaculture (“Subsecretaría de Pesca y Acuicultura”, SUBPESCA), which reports to the Chilean Ministry of Economy, Development and Tourism. The main targets of this management practice are the conservation of natural benthic resources while maintaining and promoting their productivity and also promoting the sustainability of artisanal fishing activities by involving local fishers in active participative management. Moreover, in these TURFs, artisanal fishers can implement small-scale aquaculture activities.

According to the last reports, up to October 2024, along the Chilean coasts, a total of 499 fisher organizations with recognized TURFs were reported [70], reaching a benthic resources production of 17,100 tonnes [71]. The main products extracted in these areas are algae and mollusks. Most artisanal fishing activities are reported in the Los Lagos region, in the south of Chile, with the community reaching more than 36,000 artisanal fishers at the end of 2023 [68]. Along the coast of this region, there are almost 296 TURFs [72]. For a long period, *E. megalocyathus* was confused with *O. mimus*, which lives in the coasts of Northern Chile, thus confusing the real landing values [64]. Rocha and Vega report that *E. megalocyathus* is fished south of the Ñuble region (36°00′ lat S) [73]. According to the last available data from SERNAPESCA, in the Los Lagos region, almost 571 tons of red octopus were landed in 2023 [13].

The institution of TURFs was first proposed in response to the overexploitation problem of the muricide gastropod *Concholepas concholepas*, locally known as “Loco” and worldwide as “Chilean abalone”, which represents one of the most valuable marine products of Chilean coasts. Fishers realized the rapid decline in “Loco” populations in 1990, and in the same year, Chilean legislation instituted TURFs to apply a new experimental modality of rational and sustainable management of benthic marine resources. Galarza and Kámiche Zegarra [74] report how fishing often exemplifies the so-known “Tragedy of the Commons” [75], where unrestricted access to a natural resource leads to its overexploitation and potential depletion, as individual fishers use it without awareness of being part of a system and prioritize immediate gains over long-term sustainability, ultimately threatening long-term viability of fish stocks. To overcome this problem, in 1990, Ostrom proposed increasing the capacities and responsibilities of individuals to promote cooperation and new solutions that lead to resource preservation [76]. The mandatory management plans for TURFs are based on this fundamental principle. Since the establishment of the first TURF in 1997, a consistent upward trend has resulted in the current total of 673 TURFs [72]. Early evidence of the effectiveness of this organizational structure was observed in the restoration of “Loco” wild populations, which had been overexploited for extended periods [77]. The establishment of TURFs has significantly contributed to increasing the “Loco” population within managed areas, and over the years of experience, other species have been added, such as the Patagonian red octopus *E. megalocyathus*. Other species, such as the *Gracilaria* sp. algae, have also benefited from the implementation of TURFs, although their effectiveness is not uniform across all benthic resources. Pascual-Fernández et al. report the effectiveness of participative governance in small-scale fisheries in Spain, highlighting how localized management plans for the octopus fishery, based on fish stock assessments, have helped to improve the sustainability of this sector [78].

### 3.3. Internal Governance, Decision-Making Process, and Conflict Resolution

In Chile, TURFs grant exclusive seabed access to legally constituted artisanal fishing organizations for the sustainable exploitation of benthic resources such as sea urchins, “Loco”, and octopuses. Local governance is exercised by artisanal unions, cooperatives, or associations that hold tenure over specific coastal areas, mainly composed of male members.

Decision-making is typically conducted through the Partners’ Assembly, which is the primary deliberative body of each organization, where resolutions are approved by a simple majority (50% + 1). This assembly determines key operational elements, including diver participation, harvest schedules, and pricing strategies. A Board of Directors or Management Committee, elected from among the members, is responsible for implementing the management plan, coordinating harvest activities, and securing permits.

The core management tool is the management plan, which is a technical document that must be approved by SERNAPESCA and SUBPESCA. It defines target species, harvest methods, extraction seasons, and allowable quotas. The effectiveness of the plan is assessed annually or biannually through in situ monitoring conducted by external consulting entities (typically universities or environmental firms) hired by the organization, which must be recognized by the fisheries authority and be part of a registry approved by this entity. These technical monitoring reports are mandated by Article No. 55B of Chilean General Fishing Law (Ley General de Pesca y Acuicultura No. 18892/1989) [62].

Internal and external conflicts are common in TURFs. Internally, disputes may arise over income distribution, diver schedules, or member inclusion. Externally, tensions often involve unauthorized fishing and third-party intrusion into managed areas. To address these challenges, many organizations have adopted governance tools, such as internal regulations (clearly defining rights, roles, and sanctions) and conflict mediation through union or board meetings.

In more complex cases, external institutions such as IFOP, SERNAPESCA, or INDESPA (formally “Instituto Nacional de Desarrollo Sustentable de la Pesca Artesanal y de la Acuicultura de Pequeña Escala”) may provide technical or legal support. In certain circumstances, especially where conflicts involve public order, the Ministry of the Interior may also be involved. In areas with multiple TURFs, inter-organizational coordination is often facilitated through territorial committees or collaborative networks, sometimes with municipal support acting as a neutral facilitator or notary.

Oversight of TURF management involves a tripartite system including state agencies, the fishing organizations themselves, and external partners. Resource status is periodically assessed through technical monitoring—conducted jointly by the organization and professional consultancy groups—as a prerequisite for harvest authorization. SERNAPESCA plays a central role in overseeing plan compliance, auditing logbooks, controlling landings, and detecting unauthorized extraction.

At the community level, many unions organize voluntary patrols or contract private security to prevent illegal fishing, requiring a high investment that is deducted from their profits. Additionally, some organizations implement traceability mechanisms, such as product labeling or digital dispatch guides, to ensure legal origin and maintain the integrity of supply chains [74,77,78].

A summary of institutional organizations is provided in Table 3.

### 3.4. Positive Outcomes and Strengths of TURF Implementation in Chile

In recent years, numerous studies have analyzed the implementation and effectiveness of TURFs in Chile, particularly in relation to the regulation of benthic fisheries and the underlying political objectives. The establishment of management and exploitation areas for benthic resources (AMERBs) has proven to be an efficient tool to counteract overexploitation, leading to significant increases in both the abundance and size of managed species compared to adjacent open-access areas. More importantly, AMERBs appear to generate a spillover effect that may facilitate the natural repopulation not only of the protected species themselves but also of other associated marine organisms [79,80,81]. This has resulted in higher yields, improved product quality [64], reduced navigation and search times, and consequently increased overall economic returns and strengthened community support [79]. Furthermore, management areas offer fishers greater flexibility by allowing them to schedule their harvests to align with optimal market conditions, thus maximizing economic gains [82].

Beyond economic aspects, artisanal fishing activities are deeply embedded in local communities, preserving traditional knowledge, cultural identity, and social cohesion [64]. The implementation of TURFs has empowered fishing associations to sustainably manage benthic resources, fostering participatory governance and enhancing community self-organization [81,83]. This participatory governance structure cultivates a sense of ownership and responsibility for sustainable resource use. Collaboration with professionals from various fields (biologists, sociologists, etc.) has further improved local understanding of stock assessment and resource management practices. Communal ownership encourages cooperation among fishers, strengthens stewardship of fishing grounds, and reduces illegal extraction by outsiders, with fishers actively contributing to the enforcement and surveillance of regulations. Authors reported how the local ecological knowledge of artisanal fishers is crucial for acquiring valuable ecological and fishing information on the poorly studied sea species, effectively complementing biological data to understand their distribution and characteristics, especially given that there are infrequently captured species with lack of commercial value [84].

From an ecological perspective, fishers engaged in TURF management have reported a shift in environmental awareness, recognizing the conservation benefits derived from these practices [81,85,86]. Additionally, artisanal fisheries provide diversification opportunities through small-scale aquaculture and marine ecotourism, contributing to economic stability and reducing vulnerability to external shocks [87]. Overall, artisanal fisheries sustain local economies while simultaneously strengthening social–ecological resilience by integrating economic, cultural, and environmental dimensions.

### 3.5. Challenges and Limitations of TURF Implementation

Despite the positive outcomes and intentions behind TURFs, their implementation has faced persistent challenges. One of the most critical issues that still remains is illegal fishing. Although regulations exist, effective oversight is often lacking, and local fishing organizations must allocate significant portions of their income to surveillance activities. These are typically managed through rotational efforts among members, as stipulated in their internal operating regulations [88].

Conflicts have also arisen over access and use, particularly in areas where TURFs overlap with the interests of other economic sectors such as tourism and mining or indigenous communities. These conflicts have complicated the enforcement of regulations, necessitating the formation of local working groups to manage coastal resource use [88]. Effective control throughout the entire production chain could be a way to reduce the impacts of illegal fishing, which would be complemented by social and community-based oversight.

The effectiveness of TURFs in conserving benthic resources has been variable. While some areas have shown signs of ecological recovery and evidence of seed dispersal to adjacent open-access zones, others continue to face overfishing and lack of access control [89]. Furthermore, although policy efforts aim to support local socioeconomic development, not all communities have observed tangible improvements in income or resource access. In fact, some have struggled to adapt to new restrictions imposed by TURFs, resulting in resistance or low participation.

Co-management models, where communities are involved in decision-making processes, have yielded more positive results in terms of acceptance and participation. However, the case study of the Patagonian red octopus in the Inner Sea of Chiloe highlights the ongoing risk of overexploitation. Roa-Ureta et al. [5] attribute this to a lack of fisher cooperation and the complexity and size of management areas, which impede effective implementation. The study also notes the need to consider species-specific biological factors, such as the lower fecundity of *E. megalocyathus* compared to other managed species.

In summary, while the political intention to regulate benthic fisheries through TURFs in Chile has been largely positive, their real-world effectiveness varies. Key obstacles include insufficient monitoring, illegal fishing, and socio-territorial conflicts. To improve the impact of TURFs, particularly AMERBs, greater investment in oversight, deeper community engagement, and expanded research efforts are essential to bridge the gap between policy and practice.

## 4. Octopus Fishing Practices and the Artisanal Productive Chain in Chile

Different methods for fishing octopus are used by local artisanal fishers. The most effective and used method in Chile is the hook method, which is carried out by inserting a harpoon or hook inside rock crevices in intertidal and subtidal areas (the octopus’s main refuge) to carefully extract Patagonian red octopus (*E. megalocyathus*). This is a very selective fishing method that avoids by-catch of other species, but it does not allow for differentiation of laying female octopus from male individuals, thus affecting the reproductive capacity of the species and the survival of eggs. The eggs are susceptible to microbial infections that adhere to their bodies, resulting in death [11,90,91]. Artisanal fishers usually exploit red octopus between 1 and 48 m depth [92]. Other fishing techniques are implemented around the world, depending on various factors, including the target octopus species, environmental conditions, and local traditions and regulations. The hand recollection method, which is prevalent in tropical and subtropical areas, is practiced globally. Fishers collect octopuses by hand from flat beaches and lagoons, often using a spear or harpoon to assist in extracting the animals from their shelters [4]. Pot and trap fishing methods are employed for catching octopuses on a smaller commercial scale [93]. Pots can be constructed from various materials, including terracotta, concrete, plastic pipes, and tires. Japanese fishers utilize pots with live crab as bait and a trapdoor mechanism to capture octopuses. Pots can be used for both targeted octopus fishing and for incidental catches of the animals attempting to prey on target species in other types of fishing [4,94,95]. Trawl nets represent the second most important fishing method for octopuses [96]. However, this method yields a mixed catch of lower quality and value. Large mesh trawl nets are dragged across the seabed by a vessel, capturing octopuses in their path, with a consequent high environmental impact [4]. Fyke nets are used in some areas of the world for octopus fishing. This method is mainly used for fishing *O. vulgaris* in the eastern Mediterranean, specifically in the region of Kavala and Limenas in Greece. In these areas, fyke nets, composed of two or three net chambers and hoops, are placed in shallow coastal waters, at depths ranging from 8 to 30 m [4]. Like fyke nets, baited traps are used by artisanal fishers in the Canary Islands for *O. vulgaris* fishing practice [97,98]. The barrel longline method is primarily used for fishing Giant Pacific Octopus (*Enteroctopus dofleini*) and consists of a series of floating barrels connected to a mainline with traps or pots hanging from it. Octopuses are attracted to the bait or shelter offered by the traps and become entangled within [4]. Another method is the use of boats armed with fishing lines equipped with multiple hooks at the end. The fishing lines are baited with lures such as live or dead crabs, bivalve shells, colored stones, white plastic bags, or artificial rubber lures, which are used from the shore or small boats in shallow waters [4,15]. Finally, the use of towed rakes is a traditional method employed in some areas of Japan, where rakes are dragged across the seabed by small boats to catch octopuses hiding amongst rocks or in the sand. Together with the trawl net method, towed rakes are a technique with a high environmental impact [4]. The different fishing methods are summarized in Table 4.

**Figure 3:**
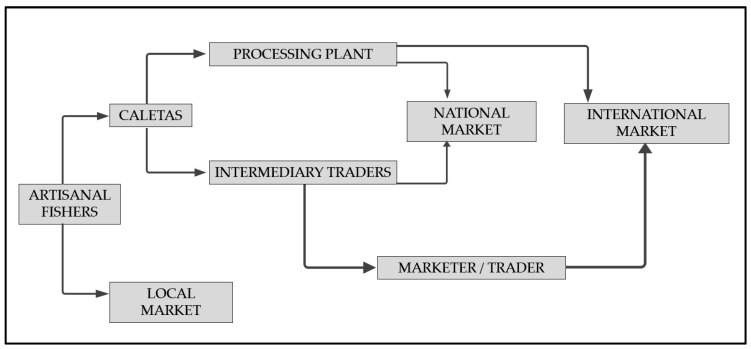
Market chain of artisanal fisheries in Chile (adapted from Benítez and Nava [131]).

Moreover, aside from the artisanal fishers (registered in RPA, as natural or juridic persons), the remaining links of the value chain include intermediaries, processors, and marketing companies, which interact directly with artisanal fishers and their organizations. These actors serve as the primary conduit through which artisanal fishery products are integrated and subsequently delivered to national and international markets (Figure 3).

Higher levels of product processing are generally associated with increased income for artisanal fishers. In cases where such value-added processing occurs, it is typically carried out by family members or individuals from the same community or fishing organization, thereby enhancing household income. It is estimated that approximately 32.5% of registered fishers engage in joint marketing activities to increase revenues, while the remaining 67.5% sell their catch to intermediaries [131].

## 5. Food Safety in the *E. megalocyathus* Artisanal Production Chain

Octopus, as a seafood product, is a significant source of high biological value protein, but it is highly perishable if appropriate safety measures are not taken. The composition of the microbial community found in the gut of octopus, which includes psychrotrophic bacteria, is influenced by the surrounding aquatic environment and may reflect its overall contamination levels [132]. As with other fishery products, cephalopods can develop different hygiene hazards during the various stages of the production chain, with possible negative repercussions for the final consumer. Contamination can occur during both the pre-harvest and post-harvest stages of seafood processing, including general food safety risks coming from bacterial contamination, such as *Salmonella* spp., *Listeria monocytogenes*, and *Aeromonas hyrophila*, spoilage and histamine-producing bacteria (*Morganella morganii*, *Klebsiella pneumoniae*, *Hafnia alvei*, *Morganella psychrotolerans*, *Acinetobacter lwoffii*, *Plesiomonas shigelloides*, *Pseudomonas* spp., *Photobacterium* spp., *Aeromonas* spp., *Vibrio* spp., *Clostridium* spp., *Proteus* spp., *Serratia* spp., *Enterobacter* spp., and *Staphylococcus* spp.) [133,134], and chemical contamination (heavy metals like mercury, cadmium, arsenic, and lead, and organic contaminants like polycyclic aromatic hydrocarbons and organochlorine pesticides) [133]. The colonization of harmful microorganisms or the presence of microbial toxins are significant health risks for consumers and can decrease seafood product quality and nutritional value. In seafood processing, the main hygiene hazards come from microbial contamination caused by poor hygienic conditions, personnel manipulation (e.g., cutting), cleaning practices, or air circulation [135]. In living or recently fished octopus, the main sources of bacterial contamination are the internal organs (mainly the digestive tract), skin, gills, and associated mucus [132]. Since the most used fishing method in artisanal fishing is harpoon, this usually leads to big wounds in the body of the octopus, which enhances the potential for contamination with environmental and gut bacteria. Evisceration has been shown to reduce microbial contamination and significantly extend the shelf life of octopus, with eviscerated specimens remaining acceptable for consumption for up to 20 days under refrigerated storage, compared to non-eviscerated specimens, which were rejected after only 10 days for *Octopus insularis* [136].

### 5.1. Pre-Harvest Algal Toxin Contamination

**Figure 4:**
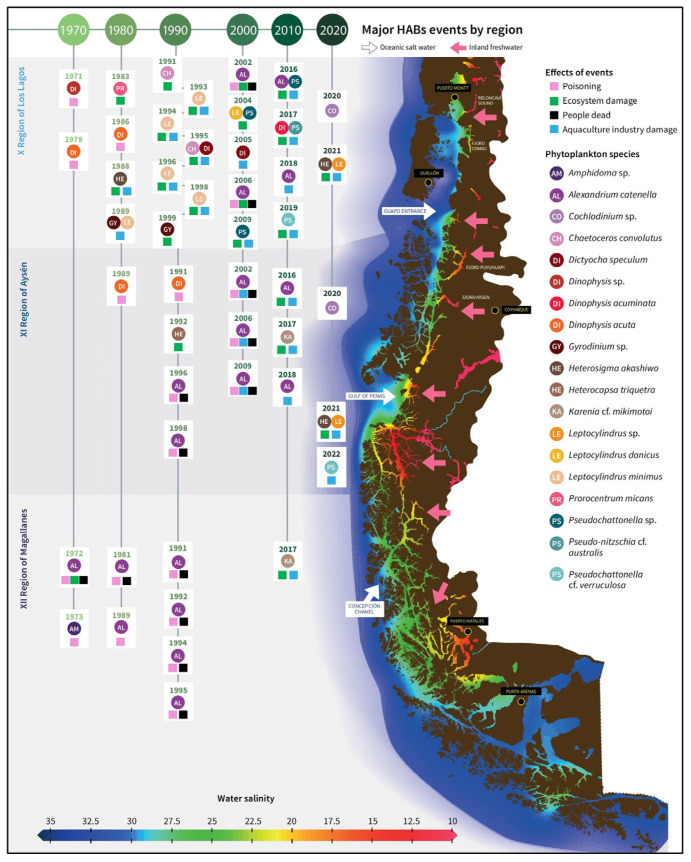
Main harmful algal bloom (HAB) events recorded (1970–2020) in southern Chilean coasts, with associated phytoplankton species reported. Different colors indicate water salinity levels (blue for higher salinity; red for lower salinity). Reproduced from [169].

Pre-harvest contamination can include pathogenic bacteria such as *Escherichia coli*, *Salmonella* spp., and *Vibrio* spp., and in some cases, algal toxins, which can be produced during certain algal blooms. The Los Lagos region, located within the Chilean Patagonian fjords, is recognized as a major hotspot significantly impacted by harmful algal blooms (HABs), with a growing trend in their temporal frequency in southern Chile and a concurrent shift of these events toward more northern regions of the country [137,138]. It hosts the highest diversity of identified HAB species in Chile, encompassing 29 species relevant to aquaculture areas [137]. Toxic HABs in this region are a paramount concern due to their capacity to transfer toxins through the food web, posing risks to human health and impacting the coastal economy, particularly aquaculture, fisheries, and tourism [139,140]. Key toxic species include the dinoflagellate *Alexandrium catenella*, responsible for Paralytic Shellfish Poisoning (PSP); various species of the genus *Dinophysis* sp. and *Protoceratium reticulatum*, associated with Diarrhetic Shellfish Poisoning (DSP); and the diatoms *Pseudo-nitzschia australis* and *P. pseudodelicatissima*, which produce domoic acid causing Amnesic Shellfish Poisoning (ASP) [141,142,143]. The spatial and temporal patterns of these events are intricate and often correlated with hydroclimatic conditions [144,145]. Significant PSP outbreaks attributed to *A. catenella* have occurred in the province of Chiloe. In 2002, an event tragically resulted in 50 human intoxications and three fatalities, alongside substantial economic losses [139,146]. Northward expansions of *A. catenella* from the Aysén region have led to outbreaks in the Los Lagos region during the late summer–early autumn of 2002 [146], 2009 [147], 2016 [148], and 2018 [149]. An intense bloom of *A. catenella* was notably recorded for the first time on the oceanic coast of the Los Lagos region in 2016 [150]. Regarding DSP, *Dinophysis* species and *P. reticulatum* are present, with *P. reticulatum* showing a positive correlation with the detection of YTXs (lipophilic toxins) in shellfish from the Los Lagos and Aysén regions [151,152,153]. While lipophilic toxin events typically occur in spring–summer, a multispecific toxic bloom in the summer of 2022 in a fjord of NW Patagonia (proximal to Los Lagos) included *P. reticulatum* and *Dinophysis acuminata* [153]. DSP toxins have been detected in the Reloncaví Estuary since 1970, and *Dinophysis acuminata* has been specifically linked to the production of PTX-2 in samples from this estuary. ASP events, primarily driven by *Pseudo-nitzschia australis*, *P. seriata,* and *P. pseudodelicatissima*, exhibit recurrent and often intense occurrences. High concentrations of domoic acid (>300 µg/g) in mussels were documented in the Los Lagos region in November 1999, and a severe event in 2014 necessitated precautionary closures [137]. More recently, the Los Lagos region experienced two prolonged events between December 2020 and January–March 2021 in the Huenquillahue and Chiloe areas. The event in Chiloe, concentrated around Isla de Quinchao, was particularly intense, with recorded values exceeding 130 µg/g of domoic acid and closures lasting 2–3 months [137]. The increasing frequency and intensity of toxic HABs in the region are partially attributed to complex interactions between environmental factors such as stratification and nutrient availability and large-scale climate change drivers [154,155], including hydroclimatic anomalies like those that characterized the summer of 2016, globally modulated by a strong ENSO (El Niño–Southern Oscillation) event, which causes lower precipitation and higher solar radiation. It is important to note that the reporting and identification of HAB events in the Los Lagos region are closely tied to areas of concentrated aquaculture. This is a possible consequence of sea water warming and high organic nutrient loads (mainly coming from nitrogen and phosphorous compounds) of anthropogenic origin, like aquaculture, agriculture, and urban or industrial wastewater [156,157,158,159]. Therefore, the total number of blooms, especially those causing minor economic impacts for artisanal fishers, may be underestimated [137]. For a geographical and historical resume of HABs in southern Chilean regions, see Figure 4.

Octopus can play a role in bioaccumulation and biomagnification of different toxic substances in the marine trophic chain [160,161]. The main source of accumulation is through the feeding of marine bivalves, which are organisms with a strong bioaccumulation potential [162]. Different authors report accumulation of algal toxins in the internal tissues of octopus (digestive gland, branchial hearts, stomach, salivary gland, and organs with excretory functions as kidneys) [163,164,165]. Particularly, the risk of toxin bioaccumulation in the digestive gland is high due to the low metabolic rate of excretion and depuration in octopus [166]. However, given that octopus is typically consumed after evisceration, the risk of bioaccumulation poses relatively low concern for food safety [160]. Relative risk can be posed by misidentification or fraud involving other toxic species [167]. The study of Oyaneder-Terrazas et al. [168] on the bioconcentration of saxitoxins in *E. megalocyathus* demonstrated a toxicity of about 3100 µg SXT-equiv 100 g^−1^, which is accumulated in fluids and the digestive gland and not detectable in the muscle that is the edible part. However, the authors warn about *E. megalocyathus* as a species that is not considered in the sanitary monitoring of saxitoxins and eventually becomes a high risk to the health of consumers of extreme ages.

### 5.2. Pre-Harvest Microbial Contamination

Unlike algal toxin contamination, which is a regional event, pre-harvest bacterial contamination tends to be more local, following direct coastal contamination. Fresh octopuses are highly susceptible to microbial contamination, which can impact quality and nutritional value. The presence of zoonotic pathogens (including *Pasteurella multocida*, *Salmonella enterica* subsp. *Typhimurium*, *Salmonella enterica* subsp. *Enteritidis*, *Chlamydia psittaci*, *Coxiella burnetii*, *Bacillus anthracis*, *Streptococcus equi*, *Pseudomonas aeruginosa*, *Escherichia coli*, *Bordetella* spp., *Staphylococcus* spp., and *Clostridium* spp.) in wetlands and watercourses flowing into the sea in the Los Lagos region of Chile raises concerns regarding potential contamination of seafood products [170]. The main source of pre-harvest octopus bacterial contamination is from total and fecal coliforms and *Vibrio* species [132,171]. Total and fecal coliforms, originating from warm-blooded animals’ gastrointestinal tracts, are recognized indicators of seafood quality and fecal contamination [172,173]. Fecal coliform concentrations in octopus can increase during the rainy season due to anthropogenic impact and favorable conditions for bacterial growth [173]. The increased freshwater flow during rainfall reduces salinity and raises water temperature, creating an ideal environment for bacterial proliferation. These conditions, combined with the presence of zoonotic pathogens in wastewater and wetlands, suggest that contaminating bacteria may flow from wetlands to the sea, contaminating fishery products and posing a risk to human health.

### 5.3. Pre-Harvest Heavy Metal Contamination

Also, the presence of heavy metals could present other health risks because cephalopods, particularly octopus, are known to potentially absorb and accumulate them in body tissues [174]. Octopus is reported as a high accumulator of heavy metals, with mainly mercury, methylmercury, arsenic, zinc, and copper being the most concentrated elements between different seafood species [175]. High arsenic concentrations, up to 48.9 ± 0.15 µg/g of wet weight, have been found in octopus [176], highlighting the concept that benthic species, which inhabit the seabed, may experience greater exposure to environmental contaminants [177]. Moreover, high arsenic concentrations have been found in *O. vulgaris* fished in the Mediterranean Sea, suggesting a relationship with anthropic activities [177], as well as in *O. mimus* from the Peruvian Pacific coasts [178]. In the Chilean austral waters, mining activities and human settlements are typically located far from the coast, suggesting a potentially low risk of heavy metal contamination in *E. megalocyathus*. However, due to high seismic and volcanic activity, ash from frequent volcanic eruptions may be a potential source of heavy metal contamination in local waters, warranting further studies to evaluate this possibility. The mechanisms of metal release involve the degassing of volatile metal compounds from the magma, physical entrainment within volcanic ash, and subsequent deposition through atmospheric fallout. Once ejected, these metals can be transported over large distances via atmospheric currents before being deposited onto terrestrial and marine ecosystems through wet or dry deposition. In coastal or island volcanic systems, direct runoff from volcanic ash deposits and surface flows after rainfall can rapidly transfer heavy metals into adjacent marine environments. Thus, volcanic activity possibly constitutes an important, although spatially and temporally variable, source of heavy metal contamination in marine ecosystems [179,180,181,182].

In cephalopods, traces of these elements are usually found in edible parts like tentacle and mantle with no risk to consumer health, but this can be an important food source to evaluate the daily dietary intake of these metals [183]. Despite this, an overestimation of health risk is common because heavy metal concentration does not reflect the bioaccessibility of these elements, which can show lower absorption during digestive processes [176]. Different authors report a lower bioaccessibility of mercury, specifically of 11% in octopus, which is one of the lowest among seafood products [175,176,184,185,186], and cadmium [187] in seafood products after cooking procedures, as the process of protein denaturation conditions the accessibility of protein–metal complexes to digestive enzymes [188,189] and leads to the formation of insoluble components [190]. Moreover, higher and potentially toxic levels of heavy metals are usually found in digestive glands [161] and internal organs [191,192], which are systematically removed during animal evisceration, with a consequent drastic reduction in potential contamination in case of human consumption. In some geographical areas, whole octopus is traditionally consumed, posing a potential health risk considering the accumulation of heavy metals in internal organs. Storelli et al. [191] reported a Spanish traditional sauce derived from whole octopus body fermentation. Assuming a weekly whole octopus intake of 100 g (comprising 96 g of mantle and 4 g of hepatopancreas), as observed in certain southern Italian regions, the authors found that high consumers of raw octopus may potentially exceed the EFSA tolerable weekly intake for cadmium, estimated at 2.5 µg/kg body weight (BW), with intake levels ranging between 2.25 and 2.84 µg/kg BW [193,194], underlining the necessity to determine implications for public health [193]. However, considering that total dietary cadmium exposure typically results from the combined intake of various foods, primarily vegetables and cereals [195,196,197], any individual risk assessment and mitigation strategy should consider the overall contribution from all relevant dietary sources.

### 5.4. Pre-Harvest Parasitic Contamination

Regarding possible parasite infestations, the presence of nematode larvae attributed to *Hysterothylacium* spp. in *Eledone* spp. (comprising *E. cirrhosa* and *E. moschata*) was reported [198,199]. The presence of *Anisakis* spp. infestation was reported in reared *O. vulgaris* populations in Galician waters, where the source of infestation was untreated, infested marine fish provided as feed (unpublished data) [200]. In their study, Picó-Durán et al. [201] report no incidence of *Anisakis* spp. in Mediterranean *O. vulgaris* from Spain, based on a parasite investigation in commercial cephalopods of the Western Mediterranean Sea.

A review of the scientific literature highlighted a significant lack of data regarding parasitic infestation in *E. megalocyathus*. In a study conducted by Garbin et al. [56], pellets from *Phalacrocorax atriceps* were analyzed, revealing the presence of *Pseudoterranova* spp. (L3 larvae) in close association with remains of *E. megalocyathus*, as determined through correspondence analysis. Nonetheless, the authors emphasized the absence of prior records documenting these anisakid larvae parasitizing this particular cephalopod species. Consequently, the role of the red octopus as an intermediate or paratenic host remains uncertain. These findings indicate a low potential health risk associated with parasitosis in octopus-derived food products.

### 5.5. Post-Harvest Microbial Contamination

Post-harvest contamination in seafood includes bacteria of public health concern, like *Escherichia coli*, *Salmonella* spp., and *Staphylococcus aureus*, as well as the proliferation of histamine-producing bacteria, including species of *Morganella* spp., *Klebsiella* spp., *Hafnia* spp., and *Photobacterium* spp. [135,202,203]. *E. coli* is an indicator of fecal contamination coming from the environment or directly from personnel manipulation operations, mostly in the last steps of the production chain, but it could be also related to fishing octopus activities in coastal areas, due to the higher water bacterial load as a consequence of major anthropogenic activities and stronger impact of environmental events such as rainy seasons [173,204].

*Salmonella* spp. is a pathogenic bacterium that can contaminate seafood mainly through unhygienic processing environments, manipulation practices, and transport [132,205], representing a health risk for final consumers [203,205,206]. Another study reported an association between domestic wastewater discharge and a great prevalence of multidrug-resistant *Salmonella* spp. in raw octopus products in Campeche, Mexico, where artisanal octopus fishing is still a common practice [203]. *Salmonella* spp. usually reach waterbodies through fecal shedding from domestic wastewater discharge [207] and can survive in the environment months or years after water contamination, depending on biotic and abiotic factors, such as water temperature, organic matter, the presence of other living organisms, and biofilm formation capacity [208]. TURFs are usually implemented far from wastewater discharge and urban activities, thus suggesting a lower risk of fecal bacterial contamination. *Staphylococcus aureus* is another pathogen reported in octopus [209]. Given it does not have marine origin, cross-contamination during manipulation is suggested because it is usually associated with human skin and mucosal microflora [210,211]. Vaiyapuri et al. [211] report the presence of Methicillin-Resistant *S. aureus* in the seafood chain because of fish handling, contaminated water, ice, or processing equipment. It is a major foodborne pathogen, which is able to tolerate a temperature range between 7 and 48 °C and minimum water activity of 0.83. This bacterium is a potential toxin producer but needs to reach a minimum concentration of 10^6^ CFU/g, which is reached only under optimum growing conditions of 37 °C and 3.5% Sodium Chloride [212,213]. Although *S. aureus* is able to grow at 7 °C, toxin production starts up to 10 °C and is very low until 20 °C [214], so foodborne illness from octopus consumption is generally low-risk when properly refrigerated [209].

### 5.6. Post-Harvest Biogenic Amine Contamination

Among chemical hazards, biogenic amines (BAs) are another potential health risk posed by octopus consumption. Biogenic amines are produced through the decarboxylation of free amino acids by microorganisms possessing decarboxylase activity, which may include both Gram-negative and Gram-positive bacteria. The formation of BAs is influenced by three principal categories of factors: (1) intrinsic properties of the raw material, such as pH and chemical composition; (2) storage and processing conditions, including whether the product is raw, dried, or cooked, as well as fermentation parameters, hygienic practices during processing, packaging methods, storage temperature, and duration; and (3) microbial contamination, particularly the presence of decarboxylase-positive bacteria [215,216,217,218]. Bacterial decarboxylation of free amino acids results in the production of potential toxic biogenic amines (e.g., histamine, cadaverine, putrescine, and tyramine), which are valuable chemical indicators for monitoring fish spoilage, as suggested by Prester et al. [219] for musky octopus (*Eledone moschata*) and other cephalopods. Psychrophilic histamine-producing bacteria can lead to the accumulation of large quantities of histamine in seafood products, highlighting the important role of *Photobacterium phosphoreum* [220]. Takahashi et al. [221] highlight the risk of histamine food poisoning associated with *Photobacterium iliopiscarium*, reporting histamine production exceeding 500 ppm of histamine even at 5 °C in broth culture trials. Histamine production is highest under acidic pH conditions and starts during the exponential growth phase. The genus *Photobacterium* is reported in *O. vulgaris* [222]. Bacterial growth is significantly influenced by temperature and overall environmental conditions, making the comparison of bacterial contamination in octopuses across geographically diverse regions difficult. The limited scientific research on bacterial contamination in *E. megalocyathus* provides insufficient evidence to fully assess the safety risks associated with this seafood product. Increased funding is essential to enhance our understanding of the health status of the red octopus and its implications for human health. One study suggests that there is a general increase in general biogenic amine content, up to 100 mg/kg, in 4 °C chilled octopus during the storage period, but with a low histamine increase of up to 9 mg/kg in the same storage conditions [223]. Gullian-Klanian et al. [202] highlight the presence of weak histamine-forming bacterial strains in the Mayan red octopus (*Octopus maya*) during refrigerated storage conditions, reporting how histamine formation is present but usually in low concentrations, which is insufficient to cause severe scombroid syndrome but useful to evaluate hygienic quality and spoiling conditions of the product. There is evidence that fresh fish sold in retail markets in Lima, Peru, had higher average histamine levels compared to fresh fish sold in wholesale markets, mainly in the afternoon, highlighting how histamine level is time-dependent [224]. Guidelines on seafood histamine content level suggest that histamine concentrations less than 5 mg/100 g of fish meat are safe for consumption, and above 5 mg there are dose-dependent toxic effects [225]. European Commission Regulation n. 2073/2005 [226], based on the Codex Alimentarius guideline “Principles for the Establishment and Application of Microbiological Criteria for Foods (CAC/GL 21-1997)”, establishes that fishery products obtained from species associated with a high histidine content (namely fish species belonging to *Scombridae, Clupeidae, Engraulidae, Coryfenidae, Pomatomidae,* and *Scombresosidae* families) must not exceed an average histamine level of 100 mg/kg (m), with a maximum limit of 200 mg/kg (M) for individual sample units, within a sampling plan that allows a maximum of two out of nine samples between the m and M values, and no samples exceeding the M value. Chilean law follows the indications of Codex Alimentarius, reporting in Article No. 324 a maximum histamine concentration of 200 mg/kg in seafood products [227].

The formation of BAs in seafood products can be effectively mitigated through the adoption of good hygienic practices throughout the entire post-harvest chain. Preventive strategies include rapid chilling of the product immediately after capture, maintaining the integrity of the cold chain (ideally at temperatures below 4 °C), and minimizing the interval of time between harvesting and processing [228]. Proper evisceration of cephalopods can further reduce microbial loads and delay spoilage processes associated with amine formation. Furthermore, the use of clean equipment, sanitized working surfaces, and hygienic handling by trained personnel significantly limits microbial contamination with decarboxylase-positive bacteria [229,230]. Modified atmosphere packaging and the application of mild acidifying agents such as acetic acid (e.g., 0.1% solutions) have also been shown to inhibit bacterial enzymatic activity responsible for amine synthesis [231]. Overall, a comprehensive strategy that combines temperature control, hygienic handling, and appropriate packaging is essential to reduce the risk of excessive BA accumulation in octopus products during storage and distribution. Considering the scarcity of studies on *E. megalocyathus* and the fact that all of these general food safety aspects have been reported in other octopus species, it is worth suggesting the allocation of more research funding to advance the understanding of the health of *E. megalocyathus* and related food safety concerns.

## 6. Possible Improvements and Sustainable Practices

A food handler is defined as any person who handles either food or surfaces that are likely to be in contact with food, such as cutlery, plates, and bowls [232]. Different factors are crucial for ensuring the safety and quality of octopus products for human consumption, particularly regarding handling, ice usage, post-fishing hygiene, and worker training. Inadequate hygiene practices during food handling are a major contributor to the spread of foodborne illnesses. This is often exacerbated by a lack of proper training for food handlers in safe food handling procedures. Once in contact with food, these pathogens can, under favorable conditions, multiply rapidly and reach levels sufficient to cause illness when consumed [233]. Due to the rapid post-mortem deterioration kinetics of octopus, their meat exhibits rapid quality deterioration, characterized by increased TVB-N levels, elevated microbial loads, reduced sensory scores, and reduced water-holding capacity; these factors collectively limit the shelf life to approximately three days. Therefore, the implementation of post-harvest handling treatments is crucial to ensure the quality and safety of the final product [234,235]. Among these treatments, cooling remains the most widely used and effective method to extend the shelf life of seafood products [236]. Nevertheless, the majority of *E. megalocyathus* fishing sites are located in remote rural areas, far from urban centers. These communities frequently face challenges such as limited access to clean water, inadequate cold chain maintenance, and the use of unsuitable work surfaces and tools [237]; moreover, they do not apply any preservation techniques immediately after capture, aside from subsequent freezing. The absence of immediate post-harvest preservation practices for *E. megalocyathus* has also been documented in Chile, both aboard artisanal fishing vessels and at landing ports [18,23]. The lack of essential infrastructure in remote fishing communities often results in subpar hygienic conditions, which directly impacts the safety and quality of seafood products. Also, proper hand hygiene is crucial in food handling to prevent the transmission of harmful microorganisms from hands to food [238].

Insufficient ice usage and breakdown in the cold chain represent critical challenges in local octopus production. Poor ice management, leading to melting and the accumulation of water at higher temperatures, creates favorable conditions for microbial proliferation and cross-contamination, compromising the safety and quality of the final product. The systematic use of ice, and particularly its implementation directly on fishing vessels using potable water and clean tanks, represents a simple yet effective strategy for mitigating food spoilage in the context of artisanal fisheries and local markets [234,239]. Preserving an uninterrupted cold chain throughout all stages of fishing, processing, and transportation is critical to inhibit bacterial proliferation and ensure the safety and quality of seafood products. Implementing immediate icing upon catch and maintaining consistent refrigeration during storage and transport significantly mitigates the risk of microbial contamination [91]. If on-boat ice production proves difficult to implement, Dima et al. [231] demonstrated that preserving octopus flesh in a 0.1% acetic acid solution effectively slows spoilage processes, extending the shelf life up to five days post-harvest while maintaining microbiological and physicochemical quality comparable to that of ice-preserved octopus. Given the limited availability of ice in octopus fishing areas, the use of a 0.1% acetic acid solution presents a simple, low-cost alternative for ensuring the quality and safety of the Patagonian red octopus, and it may be applicable to other octopus species in resource-limited contexts [231]. Official and mandatory protocols specifically addressing the effective use of ice on artisanal fishing vessels remain limited. Article No. 313 of the Chilean food regulation Decree 977/1996 [227] establishes that seafood products must be stored at appropriate temperatures to prevent decomposition and microbial growth, specifically requiring maintenance below 5 °C throughout transportation and handling. Although not mandatory, general technical guidelines, such as the Manual de Buenas Prácticas Pesqueras [240] and the FAO guide Acuicultura de pequeña escala y recursos limitados en América Latina y el Caribe [241], recommend the use of ice on board small artisanal vessels to ensure product quality and safety.

Furthermore, following Chilean Law No. 1333/1987 on Requisites of water for different usage (“Requisito de calidad de agua para diferentes usos”) [242], stricter monitoring of water quality and environmental conditions in fishing areas is crucial. Finally, another problem in the octopus fishery is the disposal of evisceration waste, which is expected to be regulated by 2028 [243]. Effective implementation requires subsidies, technical assistance, and economic support from governments and local institutions to fisher associations, providing knowledge, resources, and assistance, to incentivize sustainable practices among artisanal fishers. Training initiatives, such as seminars, manuals, and the formation of skilled personnel within associations to educate and monitor other workers, can contribute to improving and preserving food quality. Courses and classes on food safety risks can significantly enhance good manufacturing and hygiene practices in the octopus production chain. In this context, the implementation of a standardized quality index method for raw Patagonian red octopus within rural artisanal fishing communities is essential for improving product safety, preserving organoleptic properties, and enhancing market access (Table 5). Given the highly perishable nature of cephalopods and the lack of consistent post-harvest handling practices in remote areas, the development of a simple, low-cost, and reproducible QIM can assist fishers in identifying early spoilage indicators—such as alterations in odor, texture, color, or pH—that are often linked to microbial growth and the accumulation of hazardous compounds. Integrating this tool into local training initiatives enables communities to adopt evidence-based decision-making throughout the processing, storage, and marketing phases. This approach not only reduces the risk of foodborne illness but also adds value to the product by fostering adherence to hygiene and quality standards required by national and international markets. Ultimately, this initiative supports food security, economic resilience, and public health under a One Health framework, particularly in geographically isolated regions with limited access to refrigeration and infrastructure.

Another way to enhance the One Health approach within this fishing activity is the implementation of small-scale aquaculture for *E. megalocyathus*, which represents a unique opportunity to promote both sustainable production and health aspects of this valuable resource. This initiative involves pioneering land-based rearing in seawater tanks, using natural or artificial balanced diets, and actively engaging members of artisanal fishing communities. Members of the fishing union “Sindicato de Gente de Mar de Chaular” group (Chiloé province, Los Lagos region, Chile) received training on different themes, like octopus zootechnic aspects, aquaculture engineering, octopus feeding, business and management, and coaching with women and tourism, in other to promote innovative land-based small-scale octopus aquaculture systems [244]. These individuals can act as promoters of change, encouraging a transition from traditional extractive practices to more sustainable fishing methods among other artisanal fishers. Furthermore, in rural communities, the recruitment of women in this innovative activity has the potential to foster their empowerment, leadership, economic independence, social recognition, and visibilization. This is particularly significant in addressing historically entrenched hierarchical and unequal gender relations, which are socially and culturally constructed within the sex–gender system.

## 7. Conclusions

This study summarized the biology of the Patagonian red octopus (*E. megalocyathus*) and the related fishing laws and artisanal fishery practices in Chile, highlighting the significant socioeconomic role of the species in artisanal fishing communities amidst significant challenges to ecological sustainability and food safety. Different octopus fishing approaches are described. Hook fishing is the most widely practiced fishing method and, although selective, poses a threat to the reproductive success of the species due to the potential capture of brooding females; this practice could be changed by 2028 [242]. Furthermore, the red octopus production chain poses some food safety risks to final consumers. Octopus products are threatened by microbial contamination during pre- and post-harvest handling, bioaccumulation of toxins from algal blooms, and the presence of heavy metals in the marine environment. Evisceration, a common practice in octopus processing, effectively reduces the risk of contamination from toxins and heavy metals. However, inadequate hygiene and insufficient ice usage, particularly on artisanal boats in remote fishing communities, remain critical issues. To improve sustainability of this fishery sector, the positive experience of TURFs in Chile, which have proven effective in promoting the sustainable management of other species, offers a promising model for the Patagonian red octopus fishery. The integration of training programs on post-harvest handling, hygiene practices, and food safety measures within the coastal communities is crucial to ensure the long-term sustainability of this fishery. Implementation and investments in infrastructure, technical assistance, and economic support can improve hygiene and sanitary conditions in the harvesting process, ensuring access to clean water, adequate equipment, resources and training programs for fisher associations, fishing communities, and women, thus providing diversification opportunities in coastal activities. These synergistic actions will contribute to safeguarding consumer health, protecting marine ecosystems, and ensuring the economic prosperity of the communities that depend on this valuable marine resource.

## Figures and Tables

**Table 1:** Main preys and predators of the Patagonian red octopus *Enteroctopus megalocyathus* (Adapted from Ortiz et al. [17]).

Main Preys [20,42]	Main Predators
Squat lobster, *Munida subrugosa* Cod icefish species, *Patagonothoten* sp. Tractor crab, *Peltarion spinosulum* Cunningham’s triplefin, *Helocogrammoides cunninghami* *Ribeiroclinus eigenmanni* Rock crab species, *Cyrtograpsus* sp. True limpet species, *Nacella* sp. Crab eggs Octopus eggs Conspecifics	South American sea lion, *Otaria flavescens* [38,43] South American fur seal, *Arctocephalus australis* [44] Bottlenose dolphin, *Tursiops truncatus* [45] Dusky dolphin, *Lagenorhynchus obscurus* [46,47] Peale’s dolphin, *Lagenorhynchus australis* [35] Commerson’s dolphin, *Cephalorhynchus* *commersonii commersonii* [48] Beaked skate, *Dipturus chilensis* [49] Spiny dogfish, *Squalus acanthias* [36,37] Narrowmouth catshark, *Schroederichtys bivius* [36] Red cod, *Salilota australis* [50] Sandperch, *Pseudopercis semifasciata* [51,52] Argentine sea bass, *Acanthistius patachonicus* [53] Southern rockhopper penguin, *Eudyptes* *chrysocome chrysocome* [39,40] Gentoo penguin, *Pygoscelis papua* [41] Magellanic penguin, *Spheniscus magellanicus* [53] Imperial shag, *Leucocarbo atriceps* [54,55,56] Magellan cormorant, *Leucocarbo magellanicus* [57] King cormorant, *Leucocarbo albiventer* [57] Striated caracara’s, *Phalcoboenus australis* [58]

**Table 2:** Key anatomical features of *Enteroctopus megalocyathus* and *Octopus mimus* [69].

Anatomical Characteristic	*Enteroctopus megalocyathus*	*Octopus mimus*
Mantle length (max)	Up to 190 mm	Up to 190 mm
Total length (max)	Up to ~1.0 m	Up to ~1.2 m
Body weight (max)	Up to ~7 kg	Up to ~4 kg
Arm length (relative to mantle)	3.5–5× mantle length	4–6× mantle length
Arm formula ^1^	2 > 3 > 1 > 4	2 > 3 > 4 > 1
Web depth ^2^	18–23% of arm length, equal on lateral and dorsal	18–27% of arm length, deeper on lateral arms
Suckers per arm (adult)	~180–210	Up to ~330
Enlarged suckers (male)	8–10 on all arms, starting ~14th sucker (both sexes)	2–4 enlarged suckers on 2nd and 3rd arms (males)
Gills (lamellae per demibranch)	11–13	7–8
Funnel organ	W-shaped, outer limbs shorter	W-shaped, limbs equal
Radula	9 elements (7 rows of teeth + marginal plates)	9 elements (7 rows of teeth + marginal plates)
Hectocotylized arm	Right third arm, 70–90% length of opposite arm	Right third arm, ~77% length of opposite arm
Ligula length (% of hectocotylized arm)	11–22%	0.7–1.8%
Calamus length (% of ligula)	10–15%	30–60%
Spermatophore length/number	Up to 3× mantle lengths (~3 produced)	32–40% of mantle lengths/high number
Egg size/number	~17 mm/few (~1000–5000)	2–3 mm/numerous (~400,000)
Skin texture	Loose, longitudinal folds or flaps	Rugose, coarse papillae, 2–3 large over eyes
Color (live)	Reddish purple dorsally, pale ventrally	Orange to red–purple
False eye spots (ocelli)	Absent	Absent (some dark spots may appear)
Habitat depth range	5–25 m (migrates deeper in breeding season or warmer months)	0–30 m
Geographic distribution	Chilean and Argentinean Patagonia	Northern Peru to Northern Chile

^1^ Comparative length of the four arm pairs expressed numerically in decreasing order. In octopods, the non-hectocotylized arm 3 (right or left) is used in this formula. ^2^ Triangular section of the interbrachial web, which is the membranous skin connecting adjacent arms.

**Table 3:** Institutional hierarchical organizations regulating the artisanal fishing sector and TURFs (elaborated by the authors).

Level	Institution	Main Functions (Artisanal Fisheries Focus)
1	Ministry of Economy, Development and Tourism	Defines national strategies for sustainable resource use, issues decrees, allocates budgets, and oversees institutional structure.
2	SUBPESCA (Undersecretariat for Fisheries and Aquaculture)	Issues special fishing permits, sets regulations, manages the Artisanal Fishers Register and AMERBs, and defines fishing quotas and authorized species.
3	SERNAPESCA (National Fisheries and Aquaculture Service)	Maintains the Artisanal Fishers Register, oversees sanitary and traceability programs, controls landings, and ensures food safety for export.
4	IFOP (Fisheries Development Institute)	Provides scientific data and technical assessments and supports monitoring programs and stock assessments.
	INDESPA (Instituto de Desarrollo de la Pesca Artesanal y Acuicultura a Pequeña Escala)	Supports artisanal fisheries via funding, training, infrastructure, organizational strengthening, and policy implementation.
5	Advisory Councils (National and Zonal)	Provides technical input on fisheries management plans, TURFs, and policy reforms. Includes artisanal representatives.
	Scientific and Technical Committees	Advises on methodologies, sanitary standards, and policy development; uses IFOP data.
6	National Artisanal Fisheries Register	Official record of artisanal fishers, vessels, and organizations authorized to operate.
7	Artisanal Fisher Organizations	Holds rights to AMERBs, co-manages resources, represents fishers, and implements local governance structures.
8	Individual Fishers	Fishers must be registered, operate within legal norms, and can be members of organizations.

**Table 4:** Description of the main fishing methods in world octopus fisheries (elaborated by the authors).

Fishing Method	Description	Impact	Target Octopus Species	Geographical Area	References
**Hand recollection**	Operations are conducted on foot, via snorkeling, or using small boats; intertidal areas (1–30 m depth); traditional technique; and no specialized equipment.	Very selective and low-impact method.	See “Hook method”	See “Hook method”	[4,15,99]
**Hook method**	Harpoon or hook inside rock crevices (1–30 m depth); intertidal areas.	Very selective method; difficult to differentiate laying females from males; and poor quality of raw materials.	*O. cyanea* *O. hubbsorum* *O. insularis* *O. maya* *O. mimus* *C. nocturnus* *O. tehuelchus*	SW Indian Ocean NE Pacific SE Atlantic Western Central Atlantic SE Pacific SW Pacific SE Atlantic	[11,73,100,101,102,103,104,105,106,107]
**Hookah diving**	Same as the hook method, but with an air supply from a compressor on the fishing vessel (1–40 m depth).	Very selective method; difficult to differentiate laying females from males; and higher catches.	*O. mimus* *O. hubbsorum*	Ecuador Peru Chile Mexico	[108,109,110]
**Pots**	Pots of different materials that mimic refuges of octopus in sandy/muddy sea bottom; checked every 2-days (3–20 m depth).	Size selective for adults; avoids by-catch; low incidental mortalities and environmental impact; and possible plastic pollution resulting from poor management.	*O. vulgaris* *O. insularis* *O. pallidus* *O. sinensis* *A. fangsiao* *O. tetricus* *O. minor* *E. dofleini*	Mediterranean NE Atlantic Eastern Atlantic South Africa SE Atlantic Tasmania Japan Japan Western Australia Korea Alaska	[4,94,95,103,111,112,113,114,115,116,117,118,119]
**Trawl nets**	Broad-mesh net dragged by cables from fishing vessel; octopus is usually found in multispecies fishing or as by-catch (60–500 m depth depending on boat dimensions).	High impact on seabed and benthic shelf and lope communities.	*O. vulgaris* *E. moschata* *E. cirrhosa* *Amphioctopus* sp.	NW Africa (Saharan Bank) NE Atlantic NE Atlantic Gulf of Thailand	[120,121,122,123,124,125,126,127]
**Fyke nets/Baited traps**	2–3 cylindrical-/cone-shaped chambers, placed in shallow coastal waters (8–30 m depth).	Efficient and selective method; can cause occasional by-catch; low environmental impact; possible ghost-fishing when traps are lost.	*O. vulgaris* *O. minor* *E. dofleini*	Eastern Mediterranean (mainly Greece) Canary Islands NW Pacific NW Pacific	[4,95,96,97,98,127,128,129]
**Barrel longlines**	Baited barrels suspended from a long line that attracts and traps octopuses seeking shelter (20–100 m depth).	High selective method; low by-catch; and low environmental impact	*E. dofleini* *O. conispadiceus*	Japan	[4,127]
**Fishing lines**	Fishing lines with hook-equipped end that is baited with different lures (10–80 m depth).		*E. dofleini* *O. maya*	Japan Mexico (typical “*Al garete*” fishing lines)	[104,130]
**Towed rakes**	Rakes dragged over seabed to catch sand-hidden octopuses (10–30 m depth).	High environmental impact with strong seabed disturbance.	*E. dofleini*	Japan	[127]

**Table 5:** Quality index method adapted for whole raw Patagonian red octopus (*Enteroctopus megalocyathus*) (elaborated by the authors).

No.	Organoleptic Criterion	Description	Grade 1 (First Quality)	Grade 2 (Second Quality)	Grade 3 (Third/Low Quality)
1	External appearance	Shape, physical damage, and integrity	Whole octopus, no cuts or damage, and vivid and uniform color	Minor damage to arms, slightly dull	Deep cuts, missing parts, and dull colour or black spots
2	Flesh texture (raw)	Firmness, consistency to the touch	Firm to the touch, resilient	Slightly soft but maintains shape	Flaccid, falls apart when touched
3	Smell	Freshness, marine aroma	Clean marine/seaweedy smell, no strange odors	Slight smell of decomposition or ammonia	Strong, unpleasant or rotten smell
4	Mantle and arm color	Uniformity, color intensity	Bright, uniform red	Pale red or some whitish areas	Intense discoloration, black or greenish spots
5	Mucus presence	Surface cleanliness	Low presence of transparent, watery mucus	Slight presence of mucus	Abundant presence of opalescent viscous mucus or sticky surface
6	Mantle turgor	Elasticity to touch	Turgid, elastic, and bounces back when pressed	Slight dent that doesn not fully recover, low elasticity	Deforms easily, wrinkled skin
7	Eyes and suckers	Clarity, firmness	Clear and translucent eyes, black pupil; firm and well-attached suckers	Slightly opaque eye, dark red/bloody pupil; soft suckers	Sunken eyes, opaque pupil; disintegrated or missing suckers
8	Flavor (if cooked)	Taste test (optional)	Sweet, ocean-like taste, and firm texture	Mild taste, slightly soft texture	Bitter taste, disintegrated texture

## Data Availability

The data presented in this study are available on the SERNAPESCA website at https://www.sernapesca.cl/informacion-utilidad/anuarios-estadisticos-de-pesca-y-acuicultura/ (accessed on 14 May 2025), reference number [13,59,62,64,65,66,67]. These data resources are available in the public domain.
